# Preoperative MRI Parameters Predict Urinary Continence after Robot-Assisted Laparoscopic Prostatectomy in Prostatic Cancer Patients

**DOI:** 10.3390/diagnostics9030102

**Published:** 2019-08-25

**Authors:** Shinji Fukui, Yoriaki Kagebayashi, Yusuke Iemura, Yoshiaki Matsumura, Shoji Samma

**Affiliations:** Department of Urology, Nara Prefecture General Medical Center, 897-5, Shichijo-nishi machi 2 chome, Nara 630-8581, Japan

**Keywords:** bladder neck, magnetic resonance imaging, membranous urethral length, prostatectomy, urinary incontinence

## Abstract

We aimed to investigate whether preoperative MRI findings could predict the bladder neck location on postoperative cystography and recovery of urinary incontinence after robot-assisted laparoscopic radical prostatectomy (RALP). We retrospectively reviewed 270 consecutive patients who had complete preoperative data, including MRI, and underwent postoperative observation for more than three months. Preoperative MRI parameters consisted of the membranous urethral length (MUL) and pubic symphysis-prostate apex length (PAL) on sagittal images. The bladder neck location on a postoperative cystography was defined as the lowest extension of the tapering contrast medium in the bladder, and its relation to the pubic symphysis (above (higher group) and below (lower group) the middle of the pubic symphysis height) was evaluated. Those who required no pad or a safety pad were defined as being continent. PAL was significantly shorter in the higher group than that in the lower group (25.5 vs. 29.1 mm; *p* < 0.0001). The continent group at three months had a significantly longer MUL and shorter PAL than those in the incontinent group (8.1 vs. 6.7 mm; *p* < 0.05, and 26.0 vs. 28.1 mm; *p* < 0.05, respectively). Preoperative MRI parameters could predict the bladder neck location on postoperative cystograms and the recovery of urinary incontinence after RALP.

## 1. Introduction

One of the most common complications following prostatectomy for localized or locally advanced prostate cancer is urinary incontinence, which impairs the quality of life (QOL). The reported incidence of urinary incontinence after radical prostatectomy ranges from 6 to 20% [1,2]. Although more than 90% of patients achieved urinary continence by 12 months following prostatectomy, urinary incontinence shows little improvement beyond 12 months [3].

We previously reported a significant correlation between the bladder neck location on postoperative cystograms and recovery of urinary incontinence after robot-assisted laparoscopic radical prostatectomy (RALP) [4]. The location of the bladder neck above the middle of the pubic symphysis height was a significant predictor of continence on postoperative follow-up both at three months (hazard ratio (HR), 3.25; 95% confidence interval (CI), 1.86–5.66; *p* < 0.0001) and 12 months (HR 3.52; 95% CI: 1.68–7.35; *p* = 0.0008). Furthermore, the higher the bladder neck location, the earlier urinary continence was achieved after RALP.

We considered whether there is a correlation between the location of the bladder neck on postoperative cystograms and individual anatomical differences defined by preoperative MRI findings or not. In this study, we investigated the correlation between the location of the bladder neck on postoperative cystograms and preoperative MRI findings. We also evaluated whether preoperative MRI findings predict the bladder neck location on postoperative cystograms and recovery of urinary incontinence.

## 2. Materials and Methods

The institutional review board of Nara Prefecture General Medical Center approved this retrospective study (27 July 2019 approved, the institutional review board number 344).

**Figure 1:**
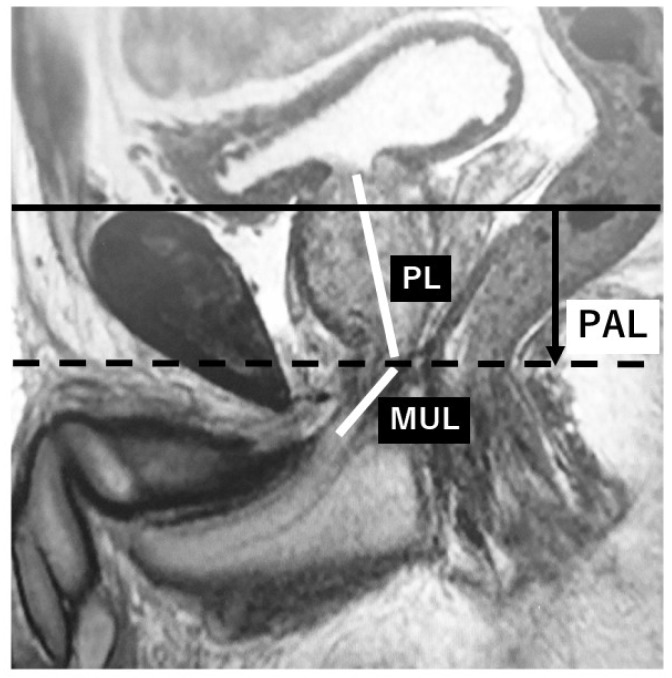
Preoperative parameters on sagittal MRI. (PL: Prostate length, MUL: Membranous urethral length, PAL: Pubic symphysis-prostate apex length, solid line: Extension line of the suprapubic ridge line, dotted line: Extension line of the prostate apical line.).

Among 285 consecutive patients with prostate cancer who underwent RALP at our hospital between March 2013 and December 2017, 270 patients were included in this study. These 270 patients had complete records of preoperative data including MRI and postoperative outcomes, and received postoperative follow-up for more than 3 months. Patients with a neurogenic bladder or end-stage kidney disease were not included. Preoperative MRI parameters consisted of the membranous urethral length (MUL), prostate length (PL), and pubic symphysis-prostate apex length (PAL) on sagittal images. PAL was defined as the distance between the extension lines of the suprapubic ridge line and the prostate apical line on sagittal MRI (Figure 1), which indicates the anatomical location of the vesico-urethral anastomosis after RALP.

We also recorded the location of the bladder neck obtained from postoperative cystograms. The bladder neck location was defined as the lowest extension of the tapering contrast medium in the bladder, and its relations with the pubic symphysis was evaluated. The bladder neck location was divided into the following 2 categories: Above and below the middle of the pubic symphysis height. The continence status was surveyed using the same questionnaire. Among information obtained at 1, 3, 6, and 12 months after RALP, data on the continence status at the 3-month follow-up were employed in this study. In analyses of urinary continence, patients who needed no pad or a safety pad were defined as being “continent”. Cystography was performed 6–8 days postoperatively in all patients.

RALP was performed using the da Vinci Si system (Intuitive Surgical, Inc., Sunnyvale, CA, USA) with a transperitoneal approach using a standard 4-armed configuration and 2 additional assistant ports.

Nerve-sparing was conducted according to the clinical stage and NCCN risk criteria. We did not carry out bladder neck preservation on a routine basis. All patients received posterior and anterior reconstructions. Van Velthoven’s running suture [5] was applied for vesico-urethral anastomosis.

Standard cystography was performed by instillation of a contrast medium into the bladder through a urethral catheter until the maximum desire to void or reaching an instillation volume of 200 mL. Cystograms were recorded in the anterior-posterior direction without catheter tension, and were interpreted by experienced urologists without information on the continence status.

The correlation between the preoperative MRI parameters (MUL, PL, and PAL) and the location of the bladder neck on postoperative cystograms was evaluated. Preoperative clinical characteristics, including the age, body mass index (BMI), NCCN risk criteria, and prostate specific antigen (PSA) value at diagnosis, and preoperative MRI parameters were also reviewed to clarify factors predicting urinary continence at the 3-month follow-up. In addition, perioperative outcomes including the operative time, console time, prostate volume, and preservation of the neurovascular bundles (none, unilateral, or bilateral) were evaluated.

The data were analyzed statistically using JMP^®^ 13 (SAS Institute Inc., Cary, NC, USA). The Mann-Whitney test, chi-square test, and Fisher’s exact test were employed to compare the data between continent and incontinent patients. Cox proportional hazards regression was used to examine variables associated with postoperative continence. A *p*-value of less than 0.05 was judged as significant.

## 3. Results

The median age of 270 patients at RALP was 69 years, and the median BMI was 23.6. The median PSA value at the diagnosis of prostate cancer was 7.9 ng/mL. The Gleason score at diagnosis was 6 in 81 patients (30%), 7 in 129 (48%), and higher than 8 in 60 (22%). The NCCN risk criteria were low in 50 patients (19%), intermediate in 144 (53%), and high in 76 (28%). The median operative and console times were 264 and 200 min, respectively. Nerve-sparing was performed in 124 patients (unilateral in 93 and bilateral in 31). A histopathological positive resection margin was observed in 68 patients (25%).

According to the postoperative cystogram findings, 174 out of 270 patients showed a bladder neck location above the middle of the pubic symphysis height (higher group), and others showed one below the middle of the pubic symphysis height (lower group). The characteristics of the higher and lower groups are presented in Table 1.

There was no significant difference between the groups divided according to the age, PSA at diagnosis, BMI, prostate volume, operative time, and console time. There was a significant difference between the groups regarding NCCN risk criteria proportions before RALP (*p* = 0.036). According to the MRI parameters, PAL showed significant differences between the groups. The higher group had a significantly shorter PAL than the lower group (25.51 vs. 29.09 mm, respectively; *p* < 0.001). There was no significant difference in MUL or PL. Patients with a higher bladder neck location had significantly more favorable continence levels than those with a lower location at the three-month follow-up (*p* < 0.0001).

Urinary continence at the three-month follow-up was achieved in 133 out of 270 patients (49%). The characteristics of the continent (133 patients) and incontinent (137 patients) groups at the three-month follow-up are presented in Table 2.

There was no significant difference between the groups divided according to the age, PSA at diagnosis, BMI, prostate volume, operative time, and console time. There was also no difference between the groups divided according to the type of nerve-sparing. When evaluating the relationship between the continent status and preoperative MRI parameters, MUL and PAL showed significant correlations. The continent group had a significantly longer MUL and shorter PAL than the incontinent group (8.1 vs. 6.7 mm; *p* = 0.043, and 26.0 vs. 28.1 mm; *p* = 0.037, respectively). However, PL did not show a difference. On multivariate regression analyses of the continent status at the three-month follow-up after RALP, only MUL remained as a significant predictor of continence (HR 2.13; 95% CI: 1.28–3.55; *p* = 0.04). PAL showed a tendency to be a predictor of continence, although this was not significant (HR 1.61; 95% CI: 0.96–12.68; *p* = 0.06) (Table 3).

**Figure 2:**
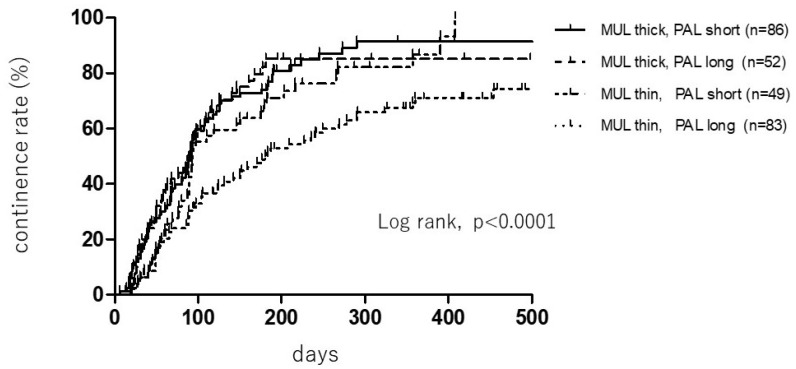
Rates of continence following robot-assisted laparoscopic radical prostatectomy according to the MRI parameters. Membranous urethral length (MUL) and pubic symphysis-prostate apex length (PAL) were defined on preoperative sagittal MRI.

According to the combination of preoperative MUL and PAL, the subjects were divided into the following four detailed categories: (1) Thicker MUL with shorter PAL, (2) thicker MUL with longer PAL, (3) thinner MUL with shorter PAL, and (4) thinner MUL with longer PAL. There was a significant correlation between the preoperative MUL and PAL, and the recovery time of urinary incontinence (the median recovery time of urinary continence was 91, 91, 93, and 180 days in the aforementioned four categories, respectively (the log rank test; *p* < 0.0001)) (Figure 2), indicating that the thinner MUL and longer PAL based on preoperative MRI were associated with a poor urinary continence status after RALP.

Patients who had a shorter PAL on preoperative MRI with a higher bladder neck position on postoperative cystograms (*n* = 99) achieved significantly earlier recovery of urinary continence than those with a shorter PAL and lower bladder neck position (*n* = 32) (the median recovery time of urinary incontinence was 89 and 179 days, respectively; the log rank test; *p* = 0.0019). However, MUL showed no significant difference (8.8 vs. 8.9 mm, respectively; *p* = 0.76).

## 4. Discussion

Urinary incontinence after RALP is still one of the major complications that leads to a poor QOL. It was reported that approximately 5% of patients after radical prostatectomy remained incontinent on a level that may impair their QOL [6]. The rates of incontinent patients after radical prostatectomy range from 6 to 20% [1,2]. The factors that can be used to predict the recovery of urinary incontinence are associated with not only surgical damage to organs regulating continence, but also with the preoperative characteristics of individual patients. The reported predictive factors are: The age [7,8,9], BMI [8], prostate volume [7], and presence of lower urinary tract dysfunction before prostatectomy [10].

In our previous report [4], we showed a significant correlation between postoperative cystogram findings and urinary continence after RALP. The bladder neck location based on postoperative cystograms was significantly correlated with the recovery of urinary incontinence. Patients with a bladder neck location above the middle of the pubic symphysis height had a significantly higher continent status at both the three- and 12-month follow-ups after RALP than those with a lower bladder neck location. Furthermore, we showed that the higher the position of the bladder neck, the earlier urinary continence was achieved (*p* < 0.0001), when the bladder neck location on postoperative cystograms was classified in detail. In this study, we evaluated the correlation between the location of the bladder neck on postoperative cystograms and preoperative MRI findings. In addition, we investigated whether preoperative MRI findings predict the bladder neck location on postoperative cystograms and urinary continence recovery.

The results of this study showed that PAL based on preoperative MRI was significantly shorter in the higher group than that in the lower group on postoperative cystograms (25.5 vs. 29.1 mm, respectively; *p* < 0.0001). This fact suggests that the anatomical location of the vesico-urethral anastomosis site, i.e., the bladder neck location based on postoperative cystograms, might be discernible on preoperative sagittal MRI, and that the location of the bladder neck on postoperative cystograms can be preoperatively predicted. There was no significant correlation between the bladder neck location on postoperative cystograms and MUL based on preoperative MRI.

When evaluating the correlation between preoperative MRI parameters and urinary continence at the three-month follow-up, there were significant correlations for MUL and PAL. MUL in the continent group was significantly thicker than that in the incontinent group (8.1 vs. 6.7 mm, respectively; *p =* 0.043), and PAL was significantly shorter than that in the incontinent group (26.0 vs. 28.1 mm, respectively; *p* = 0.037). On multivariate analysis, only MUL remained as a significant predictor of continence after RALP (HR 2.13; 95% CI: 1.28–3.55; *p* = 0.04). PAL showed a tendency toward being a predictor of continence, although this was not significant (HR 1.61; 95% CI: 0.96–12.68; *p* = 0.06).

There have been several reports of a significant correlation between MUL based on preoperative MRI and urinary continence after radical prostatectomy. Paparel et al. [11] reported that pre- and postoperative MUL and the MUL loss ratio were associated with the recovery time and level of urinary continence. Hakimi et al. [12] also reported a significant correlation between MUL on preoperative MRI and the intraoperative urethral length, and urinary continence. In a systematic review and meta-analysis [13], a longer preoperative MUL was significantly and positively correlated with the recovery of continence.

Our current study confirmed significant correlations between MUL on preoperative MRI and urinary continence, and between PAL and urinary continence at the three-month follow-up. Furthermore, when preoperative MUL and PAL were divided into four detailed categories, a significant correlation was observed between the preoperative MUL and PAL and recovery time of urinary incontinence (*p* < 0.0001) (Figure 2). This indicates that a thinner MUL with longer PAL on preoperative MRI is associated with a poor urinary continence status after RALP.

In this study, we defined PAL as the distance between the extended lines of the suprapubic ridge line and the prostate apical line on sagittal MRI, which may indicate the vesico-urethral anastomosis site at prostatectomy, i.e., the bladder neck location defined on postoperative cystograms. We hypothesized that the shorter the PAL, the higher the vesico-urethral anastomosis site, and this condition subsequently leads to the earlier recovery of urinary incontinence. This study showed that the location of the bladder neck on postoperative cystograms could be predicted preoperatively by evaluating PAL using sagittal MRI. MUL was not a factor predicting the location of the bladder neck on postoperative cystograms. Furthermore, as reported previously [4], the higher the position of the bladder neck, the earlier that urinary continence was achieved (*p* < 0.001). An independent predictive factor, however, for the urinary continence status at three months after RALP was MUL based on preoperative sagittal MRI from multivariate analysis. PAL, which was a predictive factor for the location of the bladder neck on postoperative cystograms, may also be a predictive factor for urinary continence at three months, although it was not significant (*p* = 0.06). As mentioned above, there were some discrepancies in that the predictive factor for the location of the bladder neck on postoperative cystograms was not significantly correlated with the predictive factor for the three-month urinary continence status.

This study also showed that patients with a shorter PAL on preoperative MRI and higher bladder neck position on postoperative cystograms achieved significantly earlier recovery of urinary continence than those with a shorter PAL and the lower bladder neck position on postoperative cystograms (*p* = 0.0019), although there was no significant difference in MUL (*p* = 0.76).

These results can be interpreted as follows: Not only anatomical differences on preoperative MRI but also a higher anatomical location on postoperative cystograms are necessary and important to achieve early recovery of urinary incontinence. Further studies are necessary to clarify how to support and maintain the bladder neck in the anatomic retropubic location after RALP.

There were some limitations of this study. First, this was a retrospective study with a small number of patients. Second, not a single surgeon but three experienced console surgeons performed RALP in this series. This possibly influenced postoperative urinary continence because of unavoidable technical differences among surgeons. Further studies are needed to resolve these problems.

## 5. Conclusions

In conclusion, PAL based on preoperative MRI was significantly correlated with the bladder neck location on postoperative cystograms. PAL may also be a predictive factor of urinary continence at three months after RALP. MUL showed a significant correlation with urinary continence on multivariate regression analyses. Preoperative MRI parameters can be used to predict postoperative urinary continence in patients with prostate cancer.

## Figures and Tables

**Table 1:** Comparison of characteristics between upper and lower groups on postoperative cystograms after robot-assisted laparoscopic radical prostatectomy (RALP).

	Higher Group (*n* = 174)	Lower Group (*n* = 96)	*p*-Value
Age (yrs, median [IQR])	68 (65–72)	69 (65–72)	*p* = 0.21 *
PSA (ng/mL, median [IQR])	8.1(5.7–11.3)	7.4 (5.7–11.3)	*p* = 0.66 *
BMI (median [IQR])	23.9 (22.1–25.2)	23.4 (22.1–25.2)	*p* = 0.54 *
PV (mL, median [IQR])	32.0 (25.4–39.6)	31.2 (25.0–41.5)	*p* = 0.38 *
NCCN risk criteria (*n*, %)			*p* = 0.036 **
low	40 (23)	10 (10)	
intermediate	90 (52)	54 (56)	
high	44 (25)	32 (33)	
Operative time (min, median [IQR])	266 (234–298)	259 (234–298)	*p* = 0.91 *
Console time (min, median [IQR])	199 (175–238)	204 (175–238)	*p* = 0.89 *
MUL (mm, median [IQR])	7.3 (4.2–10.7)	7.3 (5.7–9.7)	*p* = 0.49 *
PAL (mm, median [IQR])	25.5 (22.3–29.8)	29.1 (26.4–31.9)	*p* < 0.0001 *
PL (mm, median [IQR])	38.0 (33.6–41.8)	37.0 (33.9–39.2)	*p* = 0.34 *
Continence at 3-month follow-up (*n*, %)	103 (60)	30 (31)	*p* < 0.0001 ***

* Mann-Whitney test, ** chi-square test, *** Fischer’s exact test. PV; prostate volume, MUL; membranous urethral length, PAL; pubic symphysis-prostate apex length, PL; prostate length.

**Table 2:** Comparison of characteristics between continent and incontinent groups at 3-month follow-up.

	Continent (*n* = 133)	Incontinent (*n* = 137)	*p*-Value
Age (yrs, median [IQR])	68 (62–72)	69 (65–71)	*p* = 0.14 *
PSA (ng/mL, median [IQR])	8.2 (5.7–10.9)	7.4 (5.7–11.3)	*p* = 0.65 *
BMI (median [IQR])	24.0 (22.3–25.7)	23.3 (21.8–25.0)	*p* = 0.33 *
PV (mL, median [IQR])	33.6 (24.4–39.1)	31.2 (25.8–41.7)	*p* = 0.84 *
Operative time (min, median [IQR])	265 (230–299)	264 (233–285)	*p* = 0.74 *
Console time (min, median [IQR])	200 (171–238)	201 (170–230)	*p* = 1.00 *
Nerve sparing (*n*, %)			*p* = 0.09 **
none	67 (50)	79 (58)	
unilateral	45 (34)	48 (35)	
bilateral	21 (16)	10 (7)	
MUL (mm, median [IQR])	8.1 (4.6–11.0)	6.7 (5.0–9.1)	*p* = 0.043 *
PAL (mm, median [IQR])	26.0 (22.6–30.2)	28.1 (23.8–30.6)	*p* = 0.037 *
PL (mm, median [IQR])	37.5 (33.1–41.5)	37.3 (34.1–40.8)	*p* = 0.98 *

* Mann-Whitney test, ** Chi-square test. PV; prostate volume, MUL; membranous urethral length, PAL; pubic symphysis-prostate apex length, PL; prostate length.

**Table 3:** Logistic multivariate regression analyses of predictive factors of postoperative continence at 3-month follow-up.

		HR	95% CI	*p*-Value
MUL (mm)	7.3>	-		
	7.3≤	2.13	1.28–3.55	*p* = 0.004
PAL (mm)	27.3≤	-		
	27.3>	1.61	0.96–12.68	*p* = 0.06
PV (mL)	34.0>	-		
	34.0≤	1.01	0.60–1.70	*p* = 0.97

HR; hazard ratio, CI; confidence interval.
